# Seroprevalence and Risk Factor for Canine Tick-Borne Disease in Urban–Rural Area in Ayacucho, Peru

**DOI:** 10.3390/tropicalmed10090271

**Published:** 2025-09-19

**Authors:** Jime Rivera Villar, Ivonne Aquino Escalante, Saúl Chuchón Martínez, Rilder Gastelú Quispe, Ruth Huamán de la Cruz, Aide Sandoval Juarez, Giovanna Mendoza Mujica, Nyshon Rojas Palomino

**Affiliations:** 1 Facultad de Ciencias Biológicas, Universidad Nacional San Cristóbal de Huamanga, Ayacucho 05001, Peru; jimi.rivera@unsch.edu.pe (J.R.V.); ivonne.aquino02@unsch.edu.pe (I.A.E.); saul.chuchon@unsch.edu.pe (S.C.M.); rilder.gastelu@unsch.edu.pe (R.G.Q.); ruth.huaman@unsch.edu.pe (R.H.d.l.C.); 2 Centro Nacional de Salud Pública, Instituto Nacional de Salud, Lima 150108, Peru; asandoval@ins.gob.pe (A.S.J.); gmendoza@ins.gob.pe (G.M.M.)

**Keywords:** *Ehrlichia canis*, *Anaplasma*, *Borrelia burgdorferi*, ticks, tick-borne diseases

## Abstract

Ehrlichiosis and anaplasmosis are endemic to tropical and subtropical regions and pose significant zoonotic threats to both human and animal health. This study aimed to detect anti-*Ehrlichia canis*, anti-*Borrelia burgdorferi*, and anti-*Anaplasma* antibodies in dogs from the rural–urban area of Huamanga, Ayacucho. The cross-sectional survey was conducted at the Facultad de Ciencias Biológicas of the Universidad Nacional de San Cristóbal de Huamanga between May and August 2023. Samples were collected via venipuncture, and antibody detection was performed using the immunochromatographic assay Anigen Rapid CaniV-4 kit. Frequencies, percentages, and statistical analyses were conducted using the SPSS^®^ software package. A total of 107 samples from dogs in the Covadonga Human Settlement were analyzed, comprising 64 (59.8%) males and 43 (40.2%) females. The majority (78.5%) were from mixed-breed dogs, while other dogs breed included Schnauzers, Pekingese, and Pitbulls. Thirty positive samples were identified, with antibodies against *Ehrlichia canis* (15.9%), *Anaplasma phagocytophilum/Anaplasma platys* (3.7%), mixed infections of *Ehrlichia canis* and *Anaplasma phagocytophilum/Anaplasma platys* (6.5%), and *Ehrlichia canis/Borrelia burgdorferi* (1.9%) detected, as well as an association between vector exposure and the presence of *Ehrlichia canis* antibodies. These findings underscore the urgent need for the implementation of integrated control strategies and enhanced surveillance programs targeting tick-borne diseases in high-risk areas, along with targeted educational campaigns to promote responsible pet ownership and preventive measures.

## 1. Introduction

Ehrlichiosis and anaplasmosis pose significant zoonotic threats, especially in tropical and subtropical regions. Several factors influence their prevalence, including social behavior, animal husbandry practices, pet ownership, transportation, interspecies contact, insufficient sanitation, and climate change, all of which favor the proliferation of hematophagous arthropod vectors [1,2] that transmit the diseases to mammals, including domestic and wild species as well as humans, through bites [3,4,5].

Lyme disease is a significant concern in North America and Europe, with annual incidence estimates of 85,000 and 300,000 cases, respectively [6], and the USA reported more than 400,000 cases of Lyme disease between 2004 and 2016, while around 40,000 cases of ehrlichiosis/anaplasmosis were reported in the same period [7] with men between 60 and 69 years of age being the population in risk [8].

Tick-borne diseases are an emerging public health concern in Latin America. Brazil, Mexico, and Colombia have reported increasing cases, some of which are associated with higher mortality rates, likely due to delayed diagnosis and a lack of timely treatment [9,10,11,12,13].

The diagnosis of tick-borne disease is challenging. Confirmatory diagnosis relies on microscopy examinations of Giemsa-stained blood smears and in vitro culture especially for *Ehrlichia canis* and *Borrelia burgdorferi*. However, although these are considered gold standard tests due to high specificity, they are time-consuming, require expertise, and are limited by low sensitivity, especially in early infections or low pathogen load. In this context, serological tests such as ELISA, immunoblot assay, Immunofluorescence assay, and Immunochromatographic assay are commonly used for antibody detection. Conversely, although they present adequate sensitivity, their accuracy is affected by factors such as the time evolution of the disease, possible cross-reactivity, and antibody persistence after the resolution of the disease [14,15,16,17,18].

In Peru, tick-borne diseases are not subject to mandatory reporting, and current knowledge is largely derived from scientific reports. Diseases such as rickettsiosis, ehrlichiosis, and Lyme disease have been reported in Lima, Cusco, Piura, Amazonas, Ayacucho, Cajamarca, Loreto, Madre de Dios, Tumbes, and Tacna [19,20,21,22,23].

Anaplasmosis was reported in San Martín, Lambayeque, and Amazonas, and similar to others tick-borne diseases, it employs hematological, serological, and molecular methods [24,25,26].

In Ayacucho, although there are limited reports about rickettsiosis [27], it is known that *Rickettsia asembonensis* circulates in this region [23]. However, its circulation, transmission, prevalence, and distribution remain unclear. Conversely, the knowledge regarding ehrlichiosis, anaplasmosis, and Lyme disease, including their species circulation, distribution, and transmission dynamics, is very limited or nonexistent in this region.

Environmental factors and socioeconomic conditions influence the spread of these emerging diseases. However, the lack of mandatory reporting, coupled with underreporting and limited public health policies, significantly hinders epidemiological surveillance [24,28].

Clinical manifestations vary depending on the causative agent. Common symptoms include fever, headache, fatigue, general malaise, myalgia, and arthralgia, potentially reducing the quality of life. Lyme disease can lead to severe complications related to immune dysregulation, such as chronic arthritis, ophthalmic issues, and even renal, neurological, and cardiac problems, depending on the location of the pathogen [22,29,30,31]. Ehrlichiosis may also result in renal, gastrointestinal, respiratory, hepatic, and cardiac complications, including neurological involvement [32]. Infections with *Anaplasma phagocytophilum* can lead to leukopenia, intermittent thrombocytopenia, renal insufficiency, and neurological issues.

The primary vectors are hematophagous arthropods, mainly ticks, which transmit a wide array of pathogens to animals and humans, including *Borrelia*, *Rickettsia*, *Anaplasma*, *Ehrlichia canis*, and *Babesia*. Transmission success depends on their life cycle, ability to feed on various hosts at different developmental stages, and transovarial and transstadial transmission from infected female ticks [33]. *Ixodes* spp. are the main vectors of Lyme disease and anaplasmosis. Species such as *Rhipicephalus sanguineus* s.s. are vectors of *Ehrlichia canis canis* and are implicated in the transmission of *Anaplasma platys* [34,35,36,37,38]. Conversely, in Peru as well as throughout Latin America, knowledge regarding the transmission of *Anaplasma phagocytophilum* remains limited. Meanwhile, in North America, *Ixodes scapularis* and *Ixodes pacificus* have been described as vectors, whereas *Ixodes spinipalpis* and *Ixodes dentatus* are recognized as competent vectors [39,40].

Tick-borne diseases necessitate control measures that facilitate early detection, particularly in domestic animals like dogs and other companion animals, which participate in disease transmission dynamics. These animals are especially important in peri-urban and rural areas, where they serve as indicators of transmission risk [25,41].

This study aimed to determine the seroprevalence of *Ehrlichia canis*, *Borrelia burgdorferi*, and *Anaplasma* in canine blood samples from the urban–rural area of Huamanga, Ayacucho Department, Peru, to assess the potential zoonotic risk of transmission.

## 2. Materials and Methods

We conducted an observational, qualitative, and cross-sectional study in the Faculty of Biological Sciences of the Universidad Nacional San Cristóbal de Huamanga, and it was approved with RD No. 091-2023-UNSCH-FCB-D.

### 2.1. Study Area and Samples

**Figure 1:**
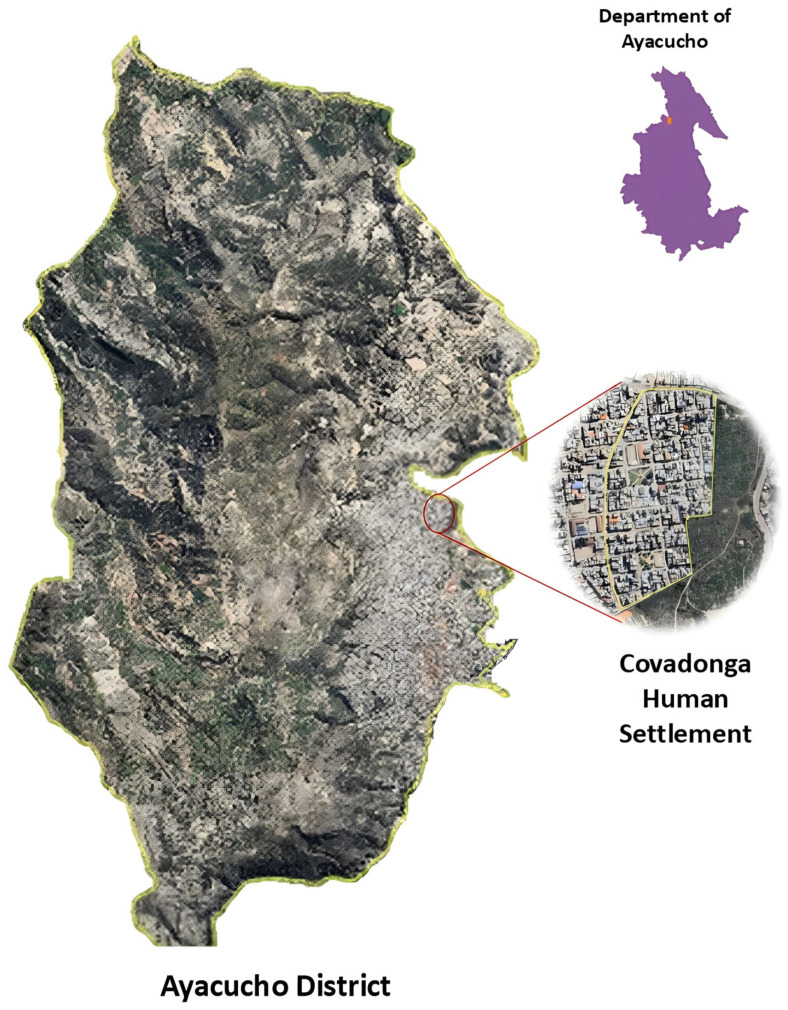
Geographical location of the samples collected for the study.

The study was conducted in the Covadonga Human Settlement (13°8′10.671″ S, 74°13′37.052″ O) located within Ayacucho District, Department of Ayacucho, Peru (Figure 1). According to the Köppen classification, this locality located at 2761 m above sea level was classified as Cwb: Subtropical Mountain climate or temperate oceanic climate with dry winters. On average, the city has a temperature of 22 °C, with November being the warmest month and July the coldest, with an average temperature of 10 °C.

The sample size was calculated using EPIDAT version 4.2 software, considering an infinite population and an estimated proportion of 64.4% of rural households in Peru that have at least one dog, with precision of 9% and a 95% confidence interval, and samples were selected through convenience sampling. The inclusion criteria required dogs to be over three months old, have resided in the study area for at least one month, and have consent from the owner or responsible caretaker.

Pregnant dogs were excluded to minimize potential stress during the collection of blood samples.

A comprehensive record of the characteristics of the dogs involved in this study was compiled, considering variables such as age, sex, general health status, presence of ectoparasites, and vaccination records.

### 2.2. Samples Collection

Blood samples were obtained by venous puncture in blood collection tubes with anticoagulant EDTA. Then, they were transported under cold chain conditions to the Genetics and Biology Laboratory of the Facultad de Ciencias Biológicas at the Universidad Nacional de San Cristobal de Huamanga.

Blood samples were centrifuged at 2500 rpm for 3 min. The supernatant was then recovered and aliquoted into 1.5 mL polypropylene conical tubes. Plasma samples were stored at −20 °C until use.

### 2.3. Antibody Detection

Antibodies were detected using the Anigen Rapid Caniv-4 Kit (BioNote Inc., Gyeonggi, Republic of Korea), an immunochromatographic assay designed for the qualitative detection of antibodies against *Ehrlichia canis*, *Borrelia burgdorferi*, *Anaplasma phagocytophilum/Anaplasma platys*, and *Dirofilaria immitis* in canine plasma. The reported sensitivities range from 93% for *Borrelia burgdorferi* and 96% for *Anaplasma phagocytophilum/Anaplasma platys* to 98% for *Ehrlichia canis* and 100% for *Dirofilaria immitis*. In terms of specificity, the assay ranges from 93% for *Borrelia burgdorferi* and 99% for *Anaplasma phagocytophilum/Anaplasma platys* detection to 100% for both *Ehrlichia canis* and *Dirofilaria immitis* [42].

Samples were processed according to the kit instructions. Briefly, all samples of each dog were processed only once; 10 µL of plasma was dispensed into sample wells, followed by 50 µL of buffer solution, and incubated at ambient temperature for 10 to 15 min.

Samples showing a specific band in the immunochromatographic test were considered positive.

### 2.4. Statistical Analysis

Contingency tables were elaborated from frequency tables and percentages of data collected from evaluated samples using an immunochromatographic test. Association analyses of variables such as age, sex, general health status, ectoparasite presence, vaccination, and antibodies against *Ehrlichia canis*, *Borrelia burgdorferi*, and *Anaplasma phagocytophilum/Anaplasma platys* were conducted using either the **χ^2^** test or Fisher’s exact test, as appropriate, with the 95% confidence interval, and a *p*-value was considered significant at *p* ≤ 0.05. This analysis was performed using SPSS^®^ version 16.0 software (SPSS Statistical Package for Social Sciences, IBM Corporation, New York, NY, USA).

## 3. Results

We collected a total of 107 blood samples from dogs in the Covadonga Human Settlement between May and July 2023. Mixed-breed dogs accounted for 78.5% of the samples, while the remaining breeds included Schnauzers, Pekingese, Pitbulls, and other breeds. Additionally, of all samples analyzed, 30 samples (28% n = 30/107) were positive in the assay, as detailed in Table 1.

Of the total samples from “mixed breed dogs”, 28 were positive (33.3%, n = 28/84). Moreover, only two samples (8.7%, n = 2/23) from “dog breed” were positive, with the categories of Pitbull and Peruvian Hairless testing positive for *Ehrlichia canis.* We found significant associations between the prevalence of tick-borne infections and dog breed (*p*-value < 0.05).

**Figure 2:**
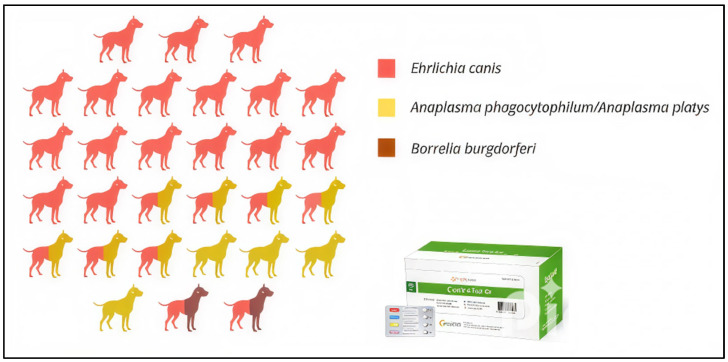
Proportion of dogs testing positive in Caniv 4 test for infections with *Ehrlichia canis*, *Anaplasma phagocytophilum/Anaplasma platys*, and *Borrelia burgdorferi*.

In addition, of all the dogs included in this study, 17 samples (15.9%, n = 17/107) were positive for *Ehrlichia canis.* Four samples (3.7%, n = 4/107) were positive for *Anaplasma phagocytophilum/Anaplasma platys*. Mixed infections were also observed, with seven samples (6.5%, n = 7/107) showing coinfection with *Ehrlichia canis* and *Anaplasma phagocytophilum/Anaplasma platys*, and two samples (1.9%, n = 2/107) testing positive for *Ehrlichia canis* and *Borrelia burgdorferi* (Table 1 and Figure 2).

Clinical assessment revealed that 56.1% of all dogs (60/107) were in good condition, while 35.5% (38/107) showed signs of regular health and 8.4% (9/107) exhibited signs of poor health. The prevalence of tick-borne disease was higher in male dogs at 35.9% (23/64), compared to 16.3% in females (7/43). Moreover, 65% of unvaccinated dogs (13/20) tested positive, whereas 33% of dogs with ectoparasite presence (29/88) also tested positive. A significant association was observed between tick infestation and the presence of *Ehrlichia canis* antibodies (*p*-value < 0.05), suggesting a link between vector exposure and infection risk, as shown in Table 2.

**Figure 3:**
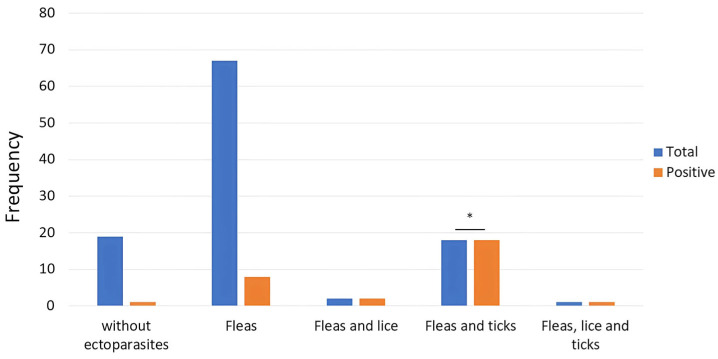
Total number of dogs according to the presence of ectoparasites (blue) and positivity in antibody detection for *Ehrlichia canis*, *Borrelia burgdorferi*, and *Anaplasma phagocytophilum/Anaplasma platys*. * Significant association (*p*-value < 0.05).

Furthermore, we observed an association between the presence of ticks and antibodies for Ehrlichia canis (*p*-value < 0.05) (Figure 3).

## 4. Discussion

The data limited to the transmission dynamics of canine ehrlichiosis, borreliosis, and anaplasmosis in Peru, particularly in the Ayacucho Department, hinders the implementation of effective disease control and prevention strategies.

In Lima, 214 samples from dogs that visited the Veterinary Clinic of the Facultad de Medicina Veterinaria at Universidad Nacional Mayor de San Marcos were analyzed using conventional PCR to determine infection rates of 5.1% for *Ehrlichia canis* and 2.8% for *Anaplasma*. Additionally, no associations were found between the variables of age, sex, or the presence of ectoparasites [43].

In other districts such as Chorrillos, La Molina, and San Juan de Miraflores, the seroprevalence of *Ehrlichia canis* varied, with rates of 19.3%, 8.7%, and 15%, respectively. This study used the immunochromatographic assay SNAP 4DX^®^, IDEXX [44]. Another study that employed Anigen Rapid Caniv-4 Kit revealed that the prevalence of *Ehrlichia canis* in a district of the capital, Rimac, was 4.3% among 5200 evaluated dogs. Meanwhile, infections caused by *Anaplasma phagocytophilum/Anaplasma platys* were 1.8%.

Additionally, this study also reported the presence of mixed infections caused by *Ehrlichia canis* and *Anaplasma phagocytophilum/Anaplasma platys* in 2.2% of the studied population [45].

In the San Martín department, a total of 65 samples from dogs in Tarapoto, Morales, Cacatachi, Banda de Shilcayo, and Juan Guerra localities were analyzed using the immunochromatographic assay SNAP^®^ 4Dx^®^ Plus, revealing a seroprevalence of 43% for *Anaplasma* [24].

Conversely, in Chiclayo, it is estimated that the prevalence reached 74.8%, which may be related to the abundant presence of the tick *Rhipicephalus sanguineus* [21]. Regarding *Anaplasma* transmission, for this city, the seroprevalence determined using the immunochromatographic test SNAP 4Dx Plus was 22.7%, although there is a report that estimates this at 34.6% [25].

Studies from countries such as Mexico reported a prevalence of 27% for *Ehrlichia canis* among 586 dogs from Chihuahua city [46], while in Mexicali Baja California, a border city between Mexico and the USA, the use of SNAP 3DX^®^ IDEXX among 54 dogs showed a prevalence of 49.3% for *Ehrlichia canis* [47].

The variability in prevalence for *Ehrlichia canis* and *Anaplasma phagocytophilum/Anaplasma platys* across different studies, including ours, could be related to a wide range of factors, such as environmental factors, like temperature, humidity, and climatic change, and socio-demographic factors, such as occupations, access healthcare, education, poverty [48,49], and even health policies.

Conversely, a total of 216 samples from dogs tested using conventional PCR for Anaplasmataceae family detection exhibited an infection rate of 40%, with 13.8% being positive for *Ehrlichia canis* and 7.4% for *Anaplasma platys*. Furthermore, that study demonstrated associations between age, sex, the presence of ectoparasites in dogs, and the owners’ socioeconomic status [50], similar to our findings.

On the other hand, several studies report differing results concerning factors associated with infection. Some identified variables such as age, tick presence [45,46] or tick exposure and dog lifestyle [51] as associated. In contrast, other studies report no association with these same variables [24,45,51], making it difficult to reach conclusions.

In our study, we found associations with sex, general health status, presence of ectoparasites, and vaccination status. However, similar to other research, age did not show a significant association with tick-borne infections. Regarding vaccination, although the categories did not appear directly related to protection against tick-borne disease, a *p*-value less than 0.001 was observed. We hypothesized that, although not directly related, vaccination status may reflect responsible pet ownership and adequate care.

For a comprehensive understanding of tick-borne diseases such as ehrlichiosis, borreliosis, and anaplasmosis, research should focus on transmission risks to both animals and humans, especially in at-risk populations such as dog owners, individuals in close contact with pets, and those involved in animal care [52,53].

The results underscore the urgent need to establish effective control strategies and measures, particularly in areas with a high transmission risk, including the implementation of epidemiological surveillance and vector control programs. Additionally, these findings highlight the necessity for targeted educational campaigns promoting responsible pet ownership and prevention strategies.

On the other hand, it is necessary to continue developing a larger number of studies and implementing diagnostic methods for bacterial zoonotic diseases that enable the early detection of these infections in both dogs and humans, using serological and molecular techniques. This approach aims to facilitate timely access to treatment and significantly improve the prompt management of patients.

The interpretation and extrapolation of these results are limited due to the cross-sectional design and potential bias from convenience sampling. Longitudinal studies are needed to establish the relationship between tick-borne exposure, vaccination status, and the transmission of tick-borne diseases in the Ayacucho Department.

## 5. Conclusions

This study demonstrates the seroprevalences of *Ehrlichia canis* and *Anaplasma phagocytophilum/Anaplasma platys*, as well as the occurrence of mixed infections of these pathogens in dogs in a settlement of Huamanga, Ayacucho, Peru. The findings are important for understanding the risk of transmission of tick-borne diseases in the region, the potential risk to humans, and the need for further studies to assess the public health impact.

To the best of our knowledge, this study represents a first effort in assessing the prevalence of tick-borne pathogens within the canine population of Ayacucho, providing essential baseline data that can serve for the design of control strategies and the implementation of preventive measures.

## Figures and Tables

**Table 1:** Seroprevalence of *Ehrlichia canis* and *Anaplasma* spp. (*Anaplasma phagocytophilum/Anaplasma platys*) in canine samples from Covadonga Human Settlement, Ayacucho, Peru.

Categories	Frequency	Prevalence				Total	*p*-Value
		*E. c.*	*A.* spp.	*E. c./A.* spp.	*E. c./B. b.*		
Mixed breed	84 (78.5%)	15 (17.9%)	4 (4.8%)	7 (8.3%)	2 (2.4%)	28 (33.3%)	0.020 *
Dog Breed	23 (21.5%)	2 (8.7%)	-	-	-	2 (8.7%)	
Total	107	17 (15.9%)	4 (3.7%)	7 (6.5%)	2 (1.9%)	30 (28%)	
Encompasses the following dog breeds							
- Schnauzer	6	-	-	-	-	-	
- Pekingese	4	-	-	-	-	-	
- Pitbull	3	1	-	-	-	1	
- German Shepherd	2	-	-	-	-	-	
- Cocker Spaniel	2	-	-	-	-	-	
- Peruvian Hairless Dog	1	1	-	-	-	1	
- Chihuahua	1	-	-	-	-	-	
- Rottweiler	1	-	-	-	-	-	
- Jack Russell Terrier	1	-	-	-	-	-	
- French bulldog	1	-	-	-	-	-	
- Shih Tzu	1	-	-	-	-	-	

*E.c. Ehrlichia canis*, *B.b. Borrelia burgdorferi*, *Anaplasma* spp: *Anaplasma phagocytophylum/Anaplasma platys*. * Significance was calculated using χ^2^ test, and a *p*-value < 0.05 was considered statistically significant.

**Table 2:** Demographic and clinical factors associated with tick-borne disease prevalence in dogs.

Variable	Category (n = 107)		Prevalence to Tick-Borne Disease	*p*-Value
Age	1 and 7 years	91 (85%)	25 (27.4%)	0.768 *
	>7 years	16 (15%)	5 (31.3%)	
Sex	Male	64 (59.8%)	23 (35.9%)	0.026 **
	Female	43 (40.2%)	7 (16.3%)	
General Status	Good	60 (56.1%)	6 (10%)	0.0000 **
	Regular	38 (35.5%)	17 (44.7%)	
	Poor	9 (8.4%)	7 (77.8%)	
Ectoparasite Presence	No	19 (17.8%)	1 (5.3%)	0.003 **
	Yes	88 (82.2%)	29 (33%)	
	Subcategories			
	Fleas	67 (62.6%)	8 (11.9%)	
	Fleas and lice	2 (1.9%)	2 (100%)	
	Fleas and ticks	18 (16.8%)	18 (100%)	
	Fleas, lice, and ticks	1 (0.9%)	1 (100%)	
Vaccination	No	20 (18.7%)	13 (65%)	0.000 **
	Yes	87 (81.3%)	17 (19.4%)	
	Subcategories			
	Distemper	11 (10.3%)	4 (36.4%)	
	Anti-rabies	53 (49.5%)	11 (20.8%)	
	Distemper + Anti-rabies	23 (21.5%)	2 (8.7%)	

* Significance was calculated using Fisher’s exact test. ** Significance was calculated using χ^2^ test. In both cases, a *p*-value < 0.05 was considered statistically significant.

## Data Availability

Study data are available from the corresponding author upon request.
